# Working Atmosphere and Job Satisfaction of Health Care Staff in Kenya: An Exploratory Study

**DOI:** 10.1155/2015/256205

**Published:** 2015-10-04

**Authors:** Katja Goetz, Michael Marx, Irmgard Marx, Marc Brodowski, Maureen Nafula, Helen Prytherch, Irene K. E. Omogi Awour, Joachim Szecsenyi

**Affiliations:** ^1^Department of General Practice and Health Services Research, University of Heidelberg, Vossstrasse 2, 69115 Heidelberg, Germany; ^2^Evaplan at the University Hospital of Heidelberg, Ringstrasse 19b, 69115 Heidelberg, Germany; ^3^AQUA-Institute for Applied Quality Improvement and Research in Health Care, Maschmühlenweg 8-10, 37073 Goettingen, Germany; ^4^Institute of Health Policy, Management and Research, NHIF Building, 2nd Floor Ragati Road, Upperhill, P.O. Box 307-00202, Nairobi, Kenya; ^5^Gesellschaft für Internationale Zusammenarbeit (GIZ), P.O. Box 41607, Nairobi 00100, Kenya

## Abstract

*Background*. Job satisfaction and working atmosphere are important for optimal health care delivery. The study aimed to document working atmosphere and job satisfaction of health care professionals in Kenya and to explore associations between job satisfaction, staff characteristics, and working atmosphere. *Methods*. Data from the integrated quality management system (IQMS) for the health sector in Kenya were used. Job satisfaction was measured with 10 items and with additional 5 items adapted to job situation in Kenya. Working atmosphere was measured with 13 item questionnaire. A stepwise linear regression analysis was performed with overall job satisfaction and working atmosphere, aspects of job satisfaction, and individual characteristics. *Results*. Out of 832 questionnaires handed out, 435 questionnaires were completed (response rate: 52.3%). Health care staff indicated high commitment to provide quality services and low levels regarding the adequacy and functionality of equipment at their work station. The aspect “support of the ministry of health” (*β* = 0.577) showed the highest score of explained variance (32.9%) regarding overall job satisfaction. *Conclusions*. IQMS which also evaluates job satisfaction and working atmosphere of health care staff provides a good opportunity for strengthening the recruitment and retention of health care staff as well as improving the provision of good quality of care.

## 1. Introduction

“At the heart of each and every health system, the workforce is central to advancing health” [1]. Health care staff are crucial for health service delivery and the provision of quality care to patients. However, constraints such as limited career opportunities, insufficient workforce, and low remuneration are known to increase the risk that health care staff migrate from their countries but also within countries such as from faith-based to public hospitals [2–5]. Unattractive working conditions of health care staff in combination with increased risk of occupational exposure or political violence have been identified as critical push factors that cause health care workers based on low- and middle-income countries to try and migrate abroad, including OECD countries [6].

The shortages of health care staff in low- and middle-income countries are dramatic. In America 24.8 health care workers per 1000 population are available, whereas, in Africa, where the burden of disease is higher, there are only 2.3 health care workers per 1,000 population [7]. These shortages have important social and economic costs to the countries concerned. It has been demonstrated that the financial loss to a country caused by the emigration of a single nurse is US$ 338,868 [6]. This is without any attempt to capture the financial value of the social costs. The out-migration of health care staff results in a loss of institutional memory and absolute shortages of much needed skills and experience. For a low-income country like Kenya it is particularly cost-intensive to continually invest in the training of health care staff and policy makers are keen to find ways to strengthen the so-called pull factors like career development, improvement of working conditions, and greater financial rewards to retain and motivate their health workforce [8]. A detailed description about the health system in Kenya is presented by the Global Health Observatory and WHO and within a report of the National Coordinating Agency for Population and Development, Kenya [9, 10].

Maternal and reproductive health outcomes are important markers of the functionality of health systems. Whilst progress has been made, achievement of the Millennium Development Goals (MDGs) for maternal and reproductive health by the year 2015 will remain elusive [11]. Weak health systems impede the performance of health care staff and prevent the delivery of quality care. Low levels of training, insufficient supervision, support, and recognition all serve to erode the motivation of health care staff [12], whilst the overall lack of staff and difficult working conditions leave health workers particularly those that provide maternal health care at high risk of burnout [13]. In the frame of the Millennium Development Goals maternal services have been prioritized and in many settings they are provided free of charge at the point of delivery which can cause demand for services to be high [14]. Moreover, maternal health has long been recognized to be an area where health workers and communities including providers of traditional health care have to work together, which makes relationships between the formal health system and the community particularly important.

Despite the important attention human resource issues have received in recent years, health workers in many low-income settings report their superiors taking little interest in their job satisfaction and work environment, although relatively simple and cost-effective steps can be taken to improve them. It was shown that recognition, responsibility, and training are the main motivational factors for retention of health workers [15]. These motivational factors are closely linked with the perception of job satisfaction. Therefore, the aim of this study was to evaluate the job satisfaction of health care staff working in maternal and reproductive health care in Kenya and to explore associations between job satisfaction, staff characteristics, and working atmosphere.

## 2. Methods

### 2.1. Design and Participants

In the frame of the collaboration between the Gesellschaft für Internationale Zusammenarbeit (GIZ) and the Kenyan Ministry of Health, a consortium including evaplan GmbH at the University of Heidelberg and the AQUA Institute in Germany and the Institute of Health Policy, Management and Research (IHPMR) in Nairobi was contracted to develop and implement an integrated quality management system (IQMS) that was initially focused on facilities providing reproductive and maternal health services. The development of the IQMS is described by Herrler et al. [16] and was inspired by the European Practice Assessment (EPA) methodology [17]. EPA represents a quality management program including validated instruments based on quality indicators for assessing practice management aiming at continuous improvement process [18].

Once the quality assessment tool IQMS had been developed it was field-tested at two facilities between January and February 2013. Public health authorities from different districts supported by GIZ (Kisumu East, Vihiga, Bondo, Butere, and Gucha) were asked to recruit interested health facilities. In total 36 health facilities responded to this call with a letter of motivation. Finally, 10 health facilities were selected to participate in this study. Inclusion criteria included the facilities provided services for the prevention of mother to child transmission and for survivors of gender-based violence.

One part of the IQMS focused upon evaluating the job satisfaction and working atmosphere of health care staff. After a pilot study, data were collected from health care staff working in 10 health facilities providing maternal and reproductive health care (district hospitals and health centres) across the aforementioned districts in Kenya.

### 2.2. Procedure and Measurement

To measure job satisfaction, aspects of working atmosphere, and other individual characteristics all participants completed a written questionnaire. Staff were encouraged to fill out the survey whilst the project coordinator was still at the facility. A collection box was left to also gather the responses from staff that were absent that day. All responses were anonymous. The questionnaire included the following items: structural questions about gender and age, how many hours a week the health care staff were contracted to work, and how many years they had worked in the facility. Job satisfaction was measured with 15 items that included a modified version of the Warr-Cook-Wall (WCW) job satisfaction scale developed by Warr et al. and additional 5 job satisfaction items for the specific job situation in Kenya [19]. The WCW-instrument measures overall job satisfaction and satisfaction with nine aspects of work which can be aligned to the theoretical background of the Two-Factor Theory found in Herzberg and colleagues [20]. These items were “amount of variety in job,” “opportunity to use abilities,” “amount of responsibility,” “recognition for work,” “freedom of working method,” “physical working condition,” “hours of work,” “income,” and “relations with colleagues.” The additional 5 job satisfaction items for the specific job situation in Kenya were “quality of materials and equipment,” “time needed to receive the results of laboratory tests,” “opportunities to attend training,” “opportunities for career advancement,” and “support from ministry of health.” Each item was rated on a 7-point Likert scale (1 = extreme dissatisfaction to 7 = extreme satisfaction). A higher overall mean score indicates higher satisfaction with job. The aspects of working atmosphere were measured by 13 items such as “the responsibilities within the team are clear,” “offering suggestions to improve the facility,” “I can speak my mind without fear of reprisal,” “the work atmosphere in the team is good,” and “communication with management is enough and frequent.” Rating options ranged from “1” (strongly disagree) to “5” (strongly agree). A higher overall mean score indicates better perception of working atmosphere.

### 2.3. Data Analysis

The analyses were performed using SPSS version 20.0 (SPSS Inc., IBM, USA). Continuous data was summarized using means and standard deviations. Categorical data was presented as frequency counts and percentages. Furthermore, a stepwise linear regression analysis was performed with overall job satisfaction as the dependent outcome variable and the different aspects of satisfaction with work, working atmosphere, and individual characteristics as the potential predictors. An alpha level of *P* < 0.05 was used for tests of statistical significance.

### 2.4. Ethical Approval

Ethical clearance was obtained from the Institutional Research Ethics Committee at Moi University, Kenya. All participants gave their informed consent. All participants received a briefing of the findings and have been involved in subsequent steps to improve the working conditions at these facilities.

## 3. Results

### 3.1. Description of the Study Sample

The 10 participating facilities can be described as follows: 3/10 were located in urban areas, 2 were located in rural areas (information from 5 facilities was missing), 4/10 were assigned as health center, 5 were assigned as district hospital (information from 1 facility was missing), and 7/10 were government-owned and financed.

Out of 832 questionnaires handed out in 10 facilities, 435 questionnaires of health care staff were returned (response rate: 52.3%).  Table 1 shows the characteristics of the participating health care staff and their professional group affiliation. The participants who returned their questionnaire had a mean age of 35.2 years (SD = 10.6). The mean of contracted working hours per week was 46.7 (SD = 21.3). The mean time period of employment at the facility was nearly 6 years (SD = 5.6). A high proportion of health care staff was nurses (27.6%).

Table 2 shows the results from the job satisfaction scale and the additional 5 job satisfaction items for the special job situation in Kenya. Health care staff in Kenya were highly satisfied with “colleagues” (mean = 5.21) and “recognition for work” (mean = 4.92) and less satisfied with “remuneration” (mean = 3.43) and “the needed materials and equipment” (mean = 3.71). Furthermore, the health care staff had a satisfactory feeling regarding their overall job satisfaction (mean = 4.87).

The different aspects of working atmosphere concerning health care staff in Kenya are presented in Table 3. They highly agree with the factors “my colleagues are committed to doing quality work” (mean = 3.99), “offering suggestions to improve the facility” (mean = 3.93), and “a good collaboration between my facility and community health workers” (mean = 3.91). The health care staff less agree with “the equipment in my work station is adequate and in good working condition” (mean = 2.57) and “a good collaboration between my facility and traditional birth attendants” (mean = 2.81).

Table 4 shows the stepwise regression analysis of the individual characteristics, working atmosphere, and satisfaction with aspects of work on overall satisfaction for health care staff in Kenya in this study. A model with 5 steps was carried out and explained more than 47% (*R* ^2^ = 0.476) of the variance on the dependent variable “overall job satisfaction.” All regression coefficients of the items in the stepwise regression analysis were statistically significant. These were four items of job satisfaction: “the support of the ministry of health,” “remuneration,” “needed materials and equipment,” and “physical working condition” and one item of working atmosphere “responsibilities within the team are clear.” In the first step of the stepwise regression analysis the item “the support of ministry of health” showed the highest score (*R* ^2^ = 0.329) of explained variance. The last step was reported in Table 4.

## 4. Discussion

In the last years there has been a growing interest in the working situation of health care staff in low- and middle-income countries [21, 22]. As there is limited published research on the relationship between job satisfaction and working atmosphere, this study contributes additional knowledge to a field of increasing importance.

Our study population presented a broad range of health care staff. The largest group was the nurses with a proportion of 27.6%. However, a high proportion of participants did not mention their professional group. There is scope important to examine the working situation of staff other than nurses in further studies. The current study gives a first impression of job satisfaction of health care staff in Kenya. In our study population we found young staff combined with a high number of contracted working hours per week and with a short time period of employment of health care staff in the facility. A possible explanation could be that positions may be vacant and only few staff are working with a high number of contracted working hours. This would reinforce the impression of the high workload being shouldered by very few staff in Kenya. In general, a high turnover rate of health care staff in Kenya can be observed [23]. Between 1999 and 2007 over 22% of the nurses in Kenya applied to out-migrate mainly to the United States or United Kingdom [6, 23]. Unattractive working condition, limited career opportunities, and weak health care systems produce dissatisfaction and demotivation with work which are reasons for leaving the country [24–26]. The results of our study showed that health care staff were least satisfied with their remuneration, material and equipment, freedom of working methods, and possibilities for career development. Different studies reported similar results about job satisfaction of health care staff in low- and middle-income countries [12, 21, 26, 27]. An improvement of the working situation of health care staff should be an important aim to strengthen a health care system [28]. Investments in training, retention, and sustenance of skilled health care workers in combination with recognition of their performance are a promising approach [29]. Furthermore, it was shown that recognition for work seems to be a strong predictor for job satisfaction of health care staff [30]. Moreover, as assumed by our data the collaboration between the facility and traditional birth attendants should be improved. It has been strongly recommended that qualified and motivated staff are available to build relationships and facilitate good cooperation with community members like in our study and traditional birth attendants [28]. Reasons for the poor cooperation between facility and traditional birth attendants should be examined in further studies.

Our data showed that relationships with colleagues have a high impact on satisfaction with job. This result does not surprise and was commonly observed internationally before [31–33]. However, collegial relationships at the workplace have been shown to mitigate an excessive workload. It was demonstrated that a high satisfaction with colleagues could reduce stress leading to more positive work environment [34]. In addition, this underlines the importance of organizing teamwork as in shifts to allow staff time-off, to build relationships with colleagues and especially with communities. It was shown that working in teams could motivate health care staff on the one hand and on the other hand it could increase effectiveness, responsiveness, and job satisfaction. Moreover, it is a cost-effective motivational factor for personnel retention [28].

Effective human resource strategies which support working condition of health care staff in low- and middle-income countries are necessary and should focuse on different levels: health system (macro level), health facility (micro level), and health workers (individual level) [28]. As a result of our study, we found a strong association between overall job satisfaction and the support by the ministry of health. It could be assumed that supporting the facilities by the ministry influences the perception of job satisfaction by staff. With the implementation of the Emergency Hiring Program supported by the government more staff could be hired, trained, and deployed [35, 36]. On the level of health facility concerning our results investing in equipment and material could be recommended. An important factor related to out-migration pertains to workforce concerns regarding occupational risks associated with the availability of safety equipment [6]. Paying attention to protecting staff from occupational risk is also known to make staff feel more appreciated [37].

The study benefited from the usage of internationally validated measures for the evaluation of job satisfaction [17]. The results of this study have to be seen in relation to maternal and reproductive health service and no general conclusions of the results regarding health care staff could be drawn. Moreover, our sample may not be representative for all facilities that provide maternal and reproductive health care in Kenya because we only involved facilities that took part in the IQMS. Participation depended upon the facility management's interest to improve the quality of care as articulated in a letter of motivation. The recruitment of health facilities was conducted by public health authorities. Therefore, a potential selection bias is indicated. Moreover, we cannot analyze the job satisfaction of each professional group separately because there was no balanced proportion between the different health care professional groups. Furthermore, we cannot analyze the data regarding facility description because there is too much information which is missing. In addition, this was an exploratory study; *P* values should be interpreted carefully. Due to the exploratory character of this study a sample size calculation could not be done. Significant results might be due to chance and will need to be confirmed in further targeted studies.

## 5. Conclusions

Job satisfaction and working atmosphere are important indicators for recruitment and retention of health care staff but also for the provision of good quality of care. Financial and nonfinancial incentives serve as motivational factors like increasing the remuneration, to invest in continuously career development or to improve the work equipment. However, these incentives will only have the desired effect, if they are introduced in supportive work environments. Therefore, an implementation of IQMS for maternal and reproductive health care in Kenya which also evaluates job satisfaction and working atmosphere of health care staff in these facilities provides a good opportunity to develop improvement strategies. Such improvement strategies should be developed with the involvement of policy makers, health facility managers, and health workers themselves.

## Figures and Tables

**Table 1:** Description of the study population.

Characteristics	Health care staff (*n* = 435)
Gender^a^	
Male	174 (40.0%)
Female	250 (57.5%)
Age, years; mean (SD)	35.2 (10.6)
Contracted working hours per week; mean (SD)	46.7 (21.3)
Time period of employment; mean (SD)	5.9 (5.6)
Type of health care staff^a^	
Nurse	120 (27.6%)
Cleaner	50 (11.5%)
Clinical officer	32 (7.4%)
Secretary/administration	23 (5.3%)
Laboratory technician	18 (4.1%)
Security	13 (3.0%)
Doctor	12 (2.8%)
Pharmacist	9 (2.1%)
Midwife	7 (1.6%)
Public health officer	3 (0.7%)
Other	94 (21.6)

^a^ *n* various due to missing data; SD standard deviation.

**Table 2:** Descriptive statistics of job satisfaction of health care staff in Kenya (*n* = 435).

Rate your satisfaction with following statements…^1^	Mean (SD)	CI (95%)
The physical working condition	4.37 (2.02)	4.12–4.56
The freedom to choose your own method of working	3.81 (2.20)	3.62–4.09
Your colleagues and fellow workers	5.21 (1.88)	5.00–5.40
The recognition of work	4.92 (2.08)	4.71–5.15
The amount of responsibility you are given	4.67 (2.20)	4.50–4.97
Your remuneration	3.43 (2.18)	3.32–3.79
The opportunity to use your abilities	4.73 (2.15)	4.54–5.00
Your hours of work	4.63 (2.31)	4.33–4.82
The amount of variety in your job	4.26 (2.13)	4.13–4.59
The materials and the equipment you need	3.71 (2.17)	3.46–3.92
The time needed to receive the results of laboratory tests	4.40 (2.10)	4.14–4.59
The opportunities to attend training	4.05 (2.37)	3.85–4.36
The opportunities for career advancement	3.88 (2.28)	3.65–4.14
The support from ministry of health	4.07 (2.17)	3.85–4.32
Taking everything into consideration, how do you feel about your job	4.87 (2.01)	4.68–5.11

^1^Range from 1 “very dissatisfied” to 7 “very satisfied.”

**Table 3:** Descriptive statistics of working atmosphere of health care staff in Kenya (*n* = 435).

Rate your agreement with following statements…^1^	Mean (SD)	CI (95%)
Supervision is provided in a supportive manner at this facility	3.71 (1.40)	3.59–3.95
Responsibilities within the team are clear	3.81 (1.37)	3.61–3.98
I feel encouraged to offer suggestions to improve the facility	3.93 (1.42)	3.81–4.17
My suggestions for improvement are taken seriously	3.17 (1.50)	3.07–3.47
The working atmosphere in the team is good	3.64 (1.36)	3.37–3.75
My colleagues are committed to doing quality work	3.99 (1.27)	3.75–4.10
At the workstations I can speak my mind without fear of reprisal	3.51 (1.54)	3.23–3.65
The ministry headquarters keep employees informed about official matters	3.31 (1.53)	3.09–3.51
The communication with management is enough and frequent	3.29 (1.48)	3.07–3.46
The equipment in my work station is adequate and in good working condition	2.57 (1.47)	2.26–2.64
There is a good collaboration between my facility and traditional birth attendants	2.81 (1.53)	2.57–2.97
There is a good collaboration between my facility and community health workers	3.91 (1.28)	3.72–4.06
There is a good collaboration between my facility and community midwives	3.22 (1.49)	3.02–3.41

^1^Range from 1 “strongly disagree” to 5 “strongly agree.”

**Table 4:** Associations of individual characteristics, working atmosphere, and satisfaction of aspects of work of health care staff on overall job satisfaction (results of stepwise linear regression analysis, under specification of standardized beta coefficient, *α* = 5%).

	Step 1	Step 2	Step 3	Step 4	Step 5
The support of the ministry of health	0.577	0.440	0.382	0.326	0.281
Your remuneration		0.313	0.284	0.218	0.198
Responsibilities within the team are clear			0.221	0.193	0.176
The materials and the equipment you need				0.193	0.170
The physical working condition					0.137

Pseudo *R* ^2^	**0.329**	**0.405**	**0.446**	**0.467**	**0.476**
